# Supplementary material for: Comparative Transcriptomic and Metabolomic Analyses Reveal the Regulatory Effect and Mechanism of Tea Extracts on the Biosynthesis of Monascus Pigments
Source: Foods. 2022 Oct 11;11(20):3159. doi: 10.3390/foods11203159 (PMC9602424; doi:10.3390/foods11203159)

Supplementary material-1

Supplementary method S1.

LC-MS analysis of components in tea extracts

Instrument model: Accela UHPLC system (Thermo Scientific, San Jose, CA) combine with Velos Pro mass spectrometer (Thermo Scientific, San Jose, CA)

Identification conditions: chromatographic column: Acquity BEH C18 column (1.7 µm, 2.1mm× 100mm); Mobile phase A: 0.1% formic acid, B: acetonitrile; Column temperature is 45 °C; Wavelength: 200~600 nm; Sample volume: 1.0 µl.

gradient elution:

0 ~ 0.25min: 99% A;

0.25~12 min: 99~50% A;

12~16 min: 50~1% A;

16~20 min: 1~99% A。

Mass spectrometry conditions: ESI negative ion mode; ESI voltage is 2.0kv; The gas flow rate of atomizer is 6.5; The offset is 80; Ion source temperature is 120 °C, desolvation temperature melting point is 450 C, atomization gas flow rate is 800 L/h and temperature is 800 C; Testing voltage: 1600v; Scanning quality range: 100 m/z ~ 1200 m/z.

Supplementary Table S1.

Primers for RT-qPCR analyzing MPs biosynthetic genes

Primers	Sequences (5′→3′)	References
β-actin F	5′-TTCGAGACCTTCAACGCCC-3′	(Wang et al., 2012)
β-actin R	5′-ACCCTCGTAGATGGGAACGA-3′	
MpPKS5 F	5′-TGTCCGACGAGTTTCTGCAA-3′	(Wang et al., 2015)
MpPKS5 R	5′-TATCAACGCTGCTTGGGCAT-3′	
MpFasA2 F	5′-ATGGATCGCCCGATCTTGTC-3′	(Wang et al., 2015)
MpFasA2 R	5′-CTTTGTCGAGTCCGCTGGAT-3′	
MpFasB2 F	5′-CCTCCAGGGATTACAACCCG-3′	(Wang et al., 2015)
MpFasB2 R	5′-ATTCAATGCCAGGTGCTCCA -3′	
mppA F	5′-TCCCGTTTCTTGGACGTGAG-3′	(Wang et al., 2015)
mppA R	5′-ACGTGCCATGGTTCTGTCTT-3′	
mppB F	5′-CGTCTCGCCCGATAACTTCA-3′	(Wang et al., 2015)
mppB R	5′-TTGACAGACGGGTCGAAGTC -3′	
mppC F	5′-CAGTCCTCGTCCCTTCCAGT -3′	(Wang et al., 2015)
mppC R	5′-CCACGGTGAAGGATGTCGAG -3′	
mppD F	5′-TCAACACGGGAGATGCTGTC-3′	(Wang et al., 2015)
mppD R	5′-GCCAAAGGACAGGAGCAGAT-3′	
mppE F	5′-CTTCCCGATGCCGTTGTGAT-3′	(Wang et al., 2015)
mppE R	5′-CGTCTCGTGGATCATCTCGT-3′	
mppR1 F	5′-TCTGCAGTATGCCATGTGGG-3′	(Wang et al., 2015)
mppR1 R	5′-ATGGCACCGTCACTTAGCTC-3′	
mppR2 F	5′-ACGAAACCCTCCATGACACC-3′	(Wang et al., 2015)
mppR2 R	5′-TGCAGACAGCCTTGTGGTAG-3′	

References

Wang, C., Chen, D., Chen, M., Wang, Y., Li, Z., Li, F., 2015. Stimulatory effects of blue light on the growth, monascin and ankaflavin production in Monascus. Biotechnol. Lett. 37, 1043–1048. doi:10.1007/s10529-014-1763-3

Wang, C., Yang, H., Chen, M., Wang, Y., Li, F., Luo, C., Zhao, S., He, D., 2012. Real-time quantitative analysis of the influence of blue light on citrinin biosynthetic gene cluster expression in Monascus. Biotechnol. Lett. 34, 1745–1748. doi:10.1007/s10529-012-0962-z

Table S2

Overview of *Monascus* transcriptome in Con and T11 groups

Sample	Clean reads	Clean bases	Error rate(%)	Q20(%)	Q30(%)	GC content(%)
Con_1	45194782	6698176143	0.026	97.62	93.18	52.6
Con_2	50446790	7518122982	0.0267	97.37	92.58	52.27
Con_3	46183040	6887952800	0.0268	97.32	92.48	52.52
T11_1	47779162	7134560832	0.0263	97.51	92.92	53.51
T11_2	44364026	6609621245	0.0262	97.55	93.02	53.56
T11_3	49019986	7303425179	0.026	97.61	93.15	53.08

Table S3

Annotated number of expressed genes

DEG Set	Total	GO	KEGG	COG	NR	Swiss-Prot	Pfam	Total_anno
Con vs. T11 transformants	9058(1)	5356 (0.5913)	3502 (0.3866)	8004 (0.8836)	8229 (0.9085)	5504 (0.6076)	6549 (0.723)	8262 (0.9121)

Table S4

Expression of key genes for *Monascus pigments* secondary metabolism

Genes Name	Gene ID	Homologous genes ID (NR)	$Log_2(FC)$
MpFasA2	BL30022203	AGL44429.1	1.18
MpFasB2	BL30015972	AGL44430.1	1.01
MpPKS5	BL30008298	ALN44200.1	1.32
mppA	BL30025317	ALN44201.1	-0.04
mppB	BL30004761	AGI63864.1	0.73
mppC	BL30011727	PSN75407.1	0.32
mppD	BL30003060	APZ73941.1	0.08
	BL30022209	APZ73942.1	0.06
mppE	BL30012672	APZ73943.1	-0.08
mppR1	BL30025323	AGL44390.1	0.43
mppR2	BL30022206	APZ73944.1	0.16

Figure. S1. Hierarchical cluster plot base on PCA

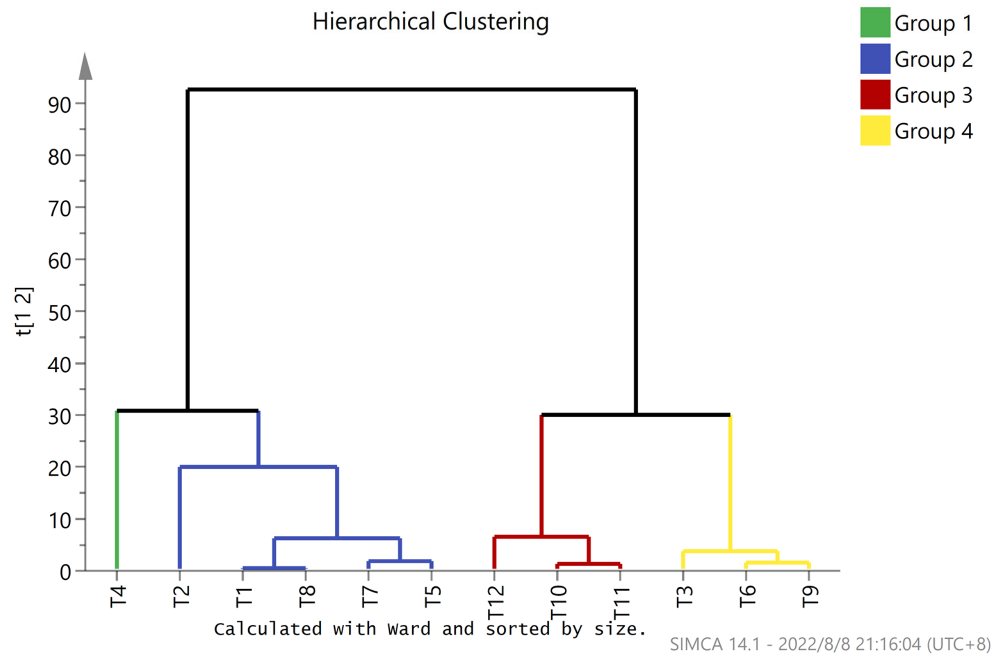

Figure. S2

Heat map of pearson correlction of genes and transcripts between Con and T11.

(A): Gene; B: transcript.

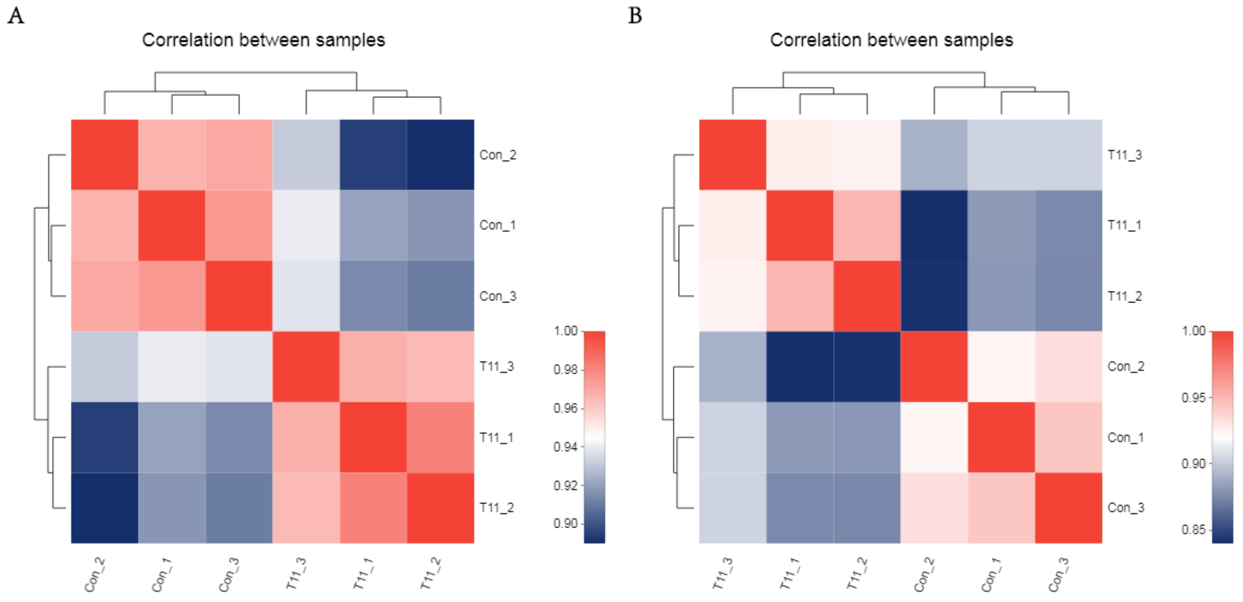

Supplement: Supplementary file 1 [file foods-11-03159-s001.zip › foods-1902255-Supplementary material/foods-1902255-Supplementary material S1.pdf]
